# Veterinarian—Chasing A Dream Job? A Comparative Survey on Wellbeing and Stress Levels among European Veterinarians between 2018 and 2023

**DOI:** 10.3390/vetsci11010048

**Published:** 2024-01-22

**Authors:** Wiebke Jansen, Lizzie Lockett, Tricia Colville, Mette Uldahl, Nancy De Briyne

**Affiliations:** 1 Federation of Veterinarians of Europe, Rue Victor Oudart 7, 1030 Brussels, Belgium; 2 Royal College of Veterinary Surgeons, The Cursitor, 38 Chancery Lane, London WC2A 1EN, UK; l.lockett@rcvs.org.uk; 3 Vets Now Emergency Limited, Penguin House, Castle Riggs, Dunfermline KY11 8SG, UK; tricia.colville@btinternet.com; 4 Vejle Hestepraksis, Fasanvej 12, 7120 Vejle, Denmark; mette@uldahl.eu

**Keywords:** veterinary, mental health, wellbeing, burnout, stress, WEMWBS, stress

## Abstract

**Simple Summary:**

Whilst recognizing the abundantly positive aspects within the different domains of the veterinary profession, the challenging socio-economic and cultural working climate has been recognized as a source of veterinary mental wellbeing issues. Comparing the results of two large-scale European surveys, stress levels and the need for medical leave due to reduced mental wellbeing remained at comparatively high levels, and mental wellbeing scores remained low. While important differences between countries were noticed, early-career veterinarians and female veterinarians were most at risk of decreased mental wellbeing all over Europe for all three indicators. Notwithstanding the increased attention that is given to veterinary wellbeing in the last decade, our results underline that major efforts remain necessary by creating more supportive and attractive workplaces that prioritize wellbeing, a good work/life balance and provide job satisfaction.

**Abstract:**

Whilst recognizing the abundantly positive aspects within the different domains of the veterinary profession, the challenging socio-economic and cultural working climate has been identified as a source of veterinary mental wellbeing issues. This mixed methods study provides an overview of the mental state of veterinarians across Europe via two cross-sectional surveys in 2018/2019 (*n* = 14,559 veterinarians) and in 2022/2023 (*n* = 12,393 veterinarians). Mental wellbeing was assessed using 3 indicators: self-reported stress levels, the need for medical leave due to reduced mental wellbeing (22% and 23%, resp., in 2018/2019 and 2022/2023) and the seven-question Warwick-Edinburgh Mental Wellbeing Scale (2018/2019: 25, 2022/2023: 24.8). In both surveys, important differences were spotlighted between countries, but early-career veterinarians and female veterinarians were most at risk of decreased mental wellbeing all over Europe for all indicators. In conclusion, stress levels and need for medical leave due to reduced mental wellbeing remained at comparatively high levels across the two surveys and standardized mental wellbeing scores remained equally low. Notwithstanding the increased attention given to veterinary wellbeing in the last decade, our results underline that major efforts remain necessary, by creating more supportive and attractive workplaces that prioritize wellbeing, a good work/life balance, and providing job satisfaction.

## 1. Introduction

Veterinary medicine is regarded as a rewarding profession by many students and veterinarians, offering many diverse career paths for adaptable professionals. Working with animals, job satisfaction in terms of challenges, and stimulus were mentioned consistently by UK veterinarians as the best parts of their job [1]. However, despite the hugely positive aspects of working in the various sectors of the veterinary profession, veterinary mental wellbeing (MWB) concerns have been linked to the challenging socio-economic and cultural working environment. Over the past decade, numerous reports have identified important stressors for veterinary professionals, such as long working hours [2,3,4,5], a lower income compared to other medical professionals [6], insufficient development opportunities, a lack of mentorship [7], challenging client communications [3,6,8,9,10,11], a demanding work–life balance [1,6], and high student debt [12,13], resulting in compassion fatigue [14,15], burnout [14,15,16,17], veterinarians feeling that they have a life not worth living [18], moral dilemmas, and other forms of stress [19]. In particular, early-career female practitioners generally seem to experience more negative stressors compared to more experienced male colleagues [2,12,20]. Publications from Europe [8,21], Australia [22,23], and the U.S. [5] have reported high stress levels, high rates of burnout, depression, and other mental illnesses, and even physical musculoskeletal disorders that are correlated with psychosocial risk factors in the veterinary profession [24]. Even though the studies suggest that the poor MWB of veterinarians differs depending on the age, gender, area of work [19,25], working hours, and relationship with clients [26], they also indicate that veterinarians have significantly poorer MWB compared to the general population [8,27]. Veterinary professionals have also been found to have one of the highest proportional mortality ratios (PMRs) for suicide of any occupation [28], around twice as high a PMR than other healthcare professions [29,30], and an overall higher PMR than the general population [29,31]. While numerous studies are available in the U.S. and some European countries, no study has been conducted so far comparing the different European countries. More recently, several studies reported that veterinary MWB has been under increasing pressure due to the global COVID-19 pandemic [32,33,34,35,36,37].

Especially in the early years of the career, emotional factors, working conditions, and business challenges might lead to disillusion, compromised enthusiasm, and burnout, and might even cause severe mental and physical health issues: In 2021, over 40% of U.S.-based practitioners, who graduated during the last 10 years, were thinking of leaving the profession, citing mental health (33%) and work–life balance (27%) as their top reasons [38]. A recent Dutch study showed that 16.8% of recently graduated practitioners left veterinary practice within five years due to excessive job demands or insufficient job resources as the most frequently cited reasons [7]. The World Health Organization (WHO) defines work-related stress as a response that people may have when presented with work demands and pressures that are not matched to their knowledge and abilities and that challenge their ability to cope [39]. This type of stress can lead to job burnout, which is a prolonged psychological response to ongoing emotional and interpersonal occupational stressors and is associated with exhaustion, cynicism, and a sense of despair [17].

While targeted surveys indicated concerns over veterinary MWB, this mixed method cross-sectional study investigated for the first time in a comparable and semi-standardized questionnaire (i) the self-reported stress levels, (ii) the need for medical leave due to reduced mental health, and (iii) the standardized MWB scores of veterinarians on a European level in 2018/2019 and 2022/2023.

## 2. Materials and Methods

This mixed methods study consisted of two cross-sectional online surveys. The STROBE (Strengthening the Reporting of Observational Studies in Epidemiology) guideline for cross-sectional studies [40] and the Checklist for Reporting Results of Internet E-Surveys (CHERRIES) [41] were used for reporting (Tables S1 and S2).

### 2.1. Surveys

Both the 2018 and 2022 survey protocol were developed by the Federation of Veterinarians of Europe (FVE). Informal beta testing of the questionnaire was carried out within FVE. Targeted social media posts, newsletters, and e-mails with an open link to the questionnaire were sent to all 38 FVE national veterinary member associations and several international organizations and corporations with a request to forward to their respective members. Participants were given extensive project information, including content, funding, contractor, and purpose. Participation was voluntary and not remunerated, and no question was compulsory, except for some of the demographic questions (e.g., gender, age) in 2018/2019. The questionnaire was online, anonymous, and issued for European veterinarians to complete between November 2018 and March 2019, and again between November 2022 and March 2023. The item-fixed questionnaire with one question per screen (59 in total) was offered in all official languages of the participating countries on veterinary demography, the demand for veterinary services, veterinary practices, working patterns of veterinarians, the future of the profession, and questions on wellbeing and stress. Some participants did not answer all questions, which led to different subtotals for each analyzed question. Questions remained editable until the submission of the questionnaire. All analyzed countries were able to recruit enough respondents in both surveys to produce country-level estimates (adjusted margin of error is ±0.80 at the 95% confidence level). The analyzed questions in this study covered assessed demographics in relation to (i) self-reported stress levels on a scale from 0 (not stressed) to 10 (very stressed) in 2018/2019 and 1 (not stressed) to 4 (very stressed) in 2022/2023, (ii) whether respondents had to take time off for medical leave due to burnout, exhaustion, compassion fatigue, or depression, and (iii) measured wellbeing scores (Table S3). For the latter, the standardized short seven-question Warwick–Edinburgh Mental Wellbeing Scale (WEMWBS—© NHS Health Scotland, The University of Warwick and University of Edinburgh, 2006, all rights reserved) was used. This scale was developed to provide a measure of MWB that is suitable for use in an adult population based on seven individual items scored on a five-point Lickert-type scale from 1 (none of the time) to 5 (all of the time). The higher the values in the score are, the higher the MWB of the individual is. Key attributes of the WEMWBS scale were its focus on the positive; its face validity among the general population, public health practitioners, and policy makers; its normal distribution in the general population, with no floor or ceiling effects; and it being validated previously with veterinary professionals [42,43,44].

### 2.2. Data Handling and Statistical Analysis

Incomplete or duplicate responses based on time stamps were removed. After this validation step, data were tabulated, processed in Microsoft^®^ Excel, and organized. An ordinal logistic regression model was used in 2022/2023 to evaluate differences between the impact of the independent variables ‘role in the veterinary profession’ (Employed full-time veterinarian (e.g., Public services, veterinary administration, practice, clinic), Employed part-time veterinarian (e.g., Public services, veterinary administration, practice, clinic), Owner/partner veterinarian/practice manager, Non-practicing veterinarian (industry), Non-practicing veterinarian (academia), and Interim/locum/freelance) on the ‘stress level’ (1, 2, 3, 4). Calculations were performed in RStudio (package stats, MASS, and dbplyr), and a *p*-value of ≤0.05 was considered significant. The comparison of cumulative WEMWB scale results was performed with an unpaired *t*-Test. A *p*-value of ≤0.05 was considered significant. Data were analyzed and plotted using MS Excel and GraphPad Prism.

## 3. Results

### 3.1. Demographics

The estimated number of active veterinarians in Europe was 309.144 in 2020 [2]. In 2018/2019, a total of 14,559 veterinary professionals coming from 30 countries completed the survey, while in 2022/2023, 12,397 veterinarians from 37 European countries did. The 2018/2019 survey mandatorily asked for age and gender (only options were female/male). The 2022/2023 survey allowed participants to select ‘other gender’ (selected 11 times) or ‘prefer not to say’ (selected 74 times). In both surveys, most respondents were female (58% and 65%, resp.) and under 44 years of age (59% and 56%). To allow for comparison, the same 27 European countries were selected based on the criterion of more than 30 respondents in both surveys (Table S4).

### 3.2. Self-Reported Stress Levels of Veterinary Professionals

**Figure 1:**
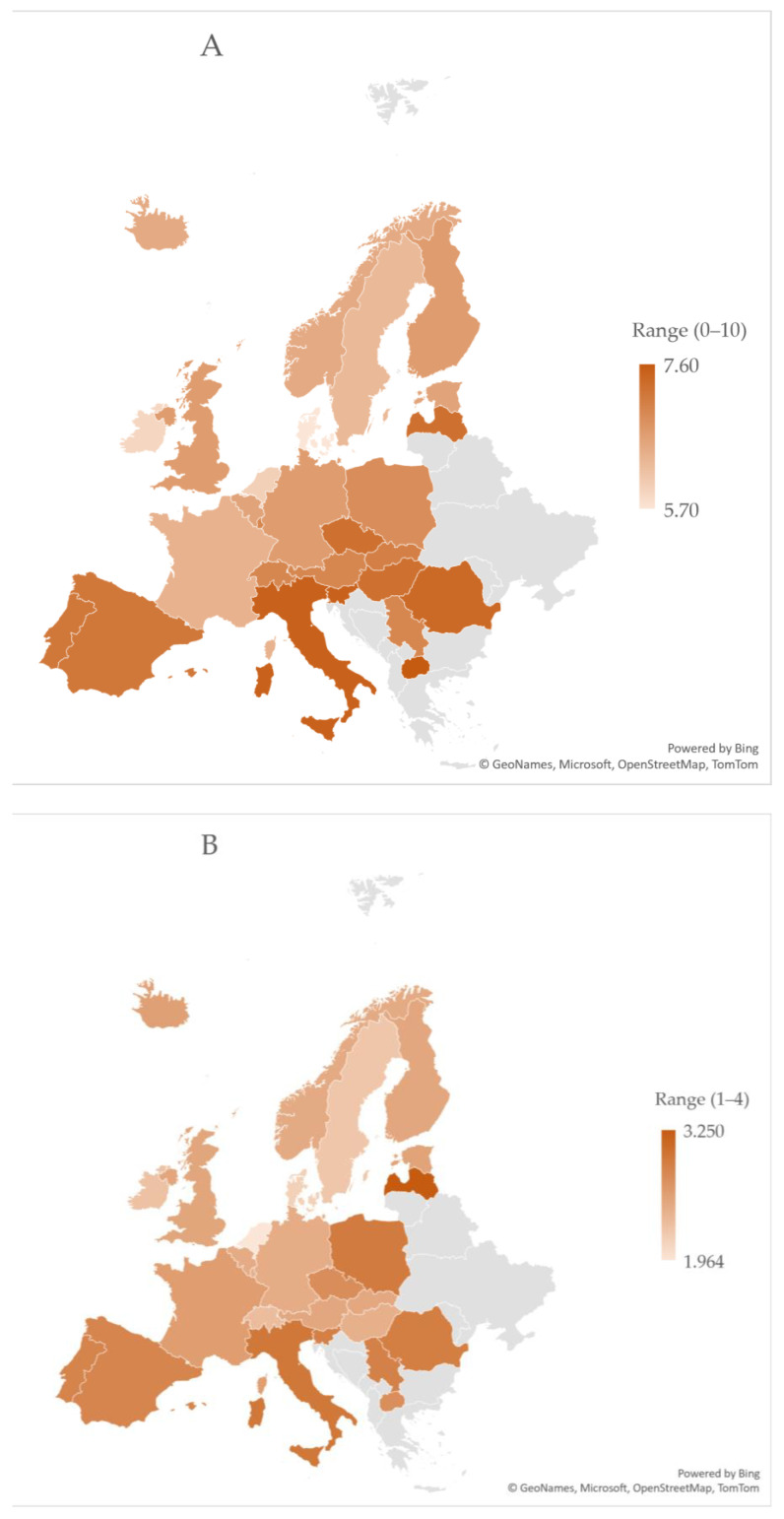
Heatmap showing veterinary self-reported stress levels at work by country. The darker the orange color is, the higher the stress level in the country is. (Panel **A**): years 2018/2019 (range: min. 0 to max. 10), (Panel **B**): years 2022/2023 (range: min. 1 to max. 4).

The average stress level for European veterinarians was 6.9 in 2018/2019 (scale of 0 lowest–10 highest) and 2.7 in 2022/2023 (scale of 1 lowest–4 highest). The self-reported levels of stress varied significantly between countries, namely, between 5.7 and 7.6 in 2018/2019 (scale of 0–10) and between 2 and 3.3 in 2022/2023 (scale of 1–4). In 2018/2019, veterinarians from North Macedonia, Italy, and Slovenia reported the highest stress levels country-wise. Those from Denmark, Ireland, and the Netherlands reported the lowest levels of stress. In 2022/2023, veterinarians from Latvia and Cyprus reported the highest levels of stress, while those from The Netherlands and Denmark reported the lowest stress levels (Figure 1).

**Figure 2:**
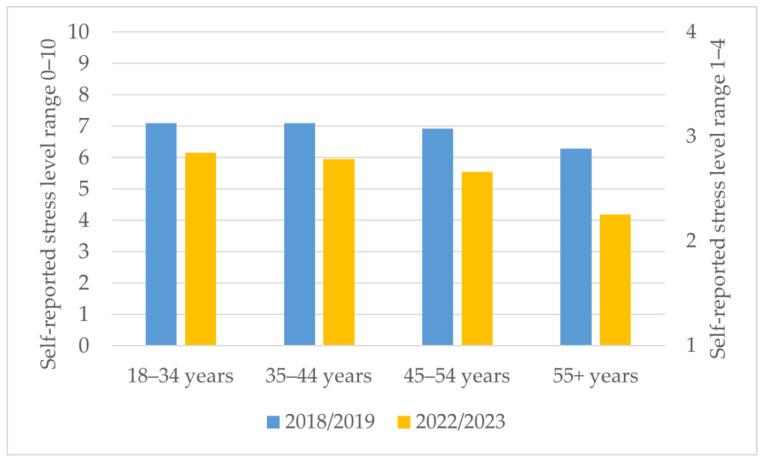
Average veterinary self-reported stress levels at work by age categories on the *y*-axis, first *x*-axis: years 2018/2019 (range: min. 0 to max. 10), secondary *x*-axis: years 2022/2023 (range: min. 1 to max. 4).

The stress levels of female and male veterinarians differed across Europe, with a few countries noting higher stress levels in male veterinarians, but with most reporting higher levels in female veterinarians. The average stress level for European female veterinarians was 7 in 2018/2019 and 2.7 in 2022/2023, and for male veterinarians, they were 6.8 and 2.5, resp. Stress levels decreased consistently in both surveys with age (Figure 2). The ordinal logistic regression showed that in 2022/2023, full-time employees (*p* < 0.001, OR 1.62, 95% CI:1.31–2.01) and practice owners and managers (*p* < 0.001, OR 1.43, 95% CI: 1.15–1.78) scored significantly higher in self-reported stress levels.

### 3.3. Medical Leave Due to Reduced Mental Health

**Figure 3:**
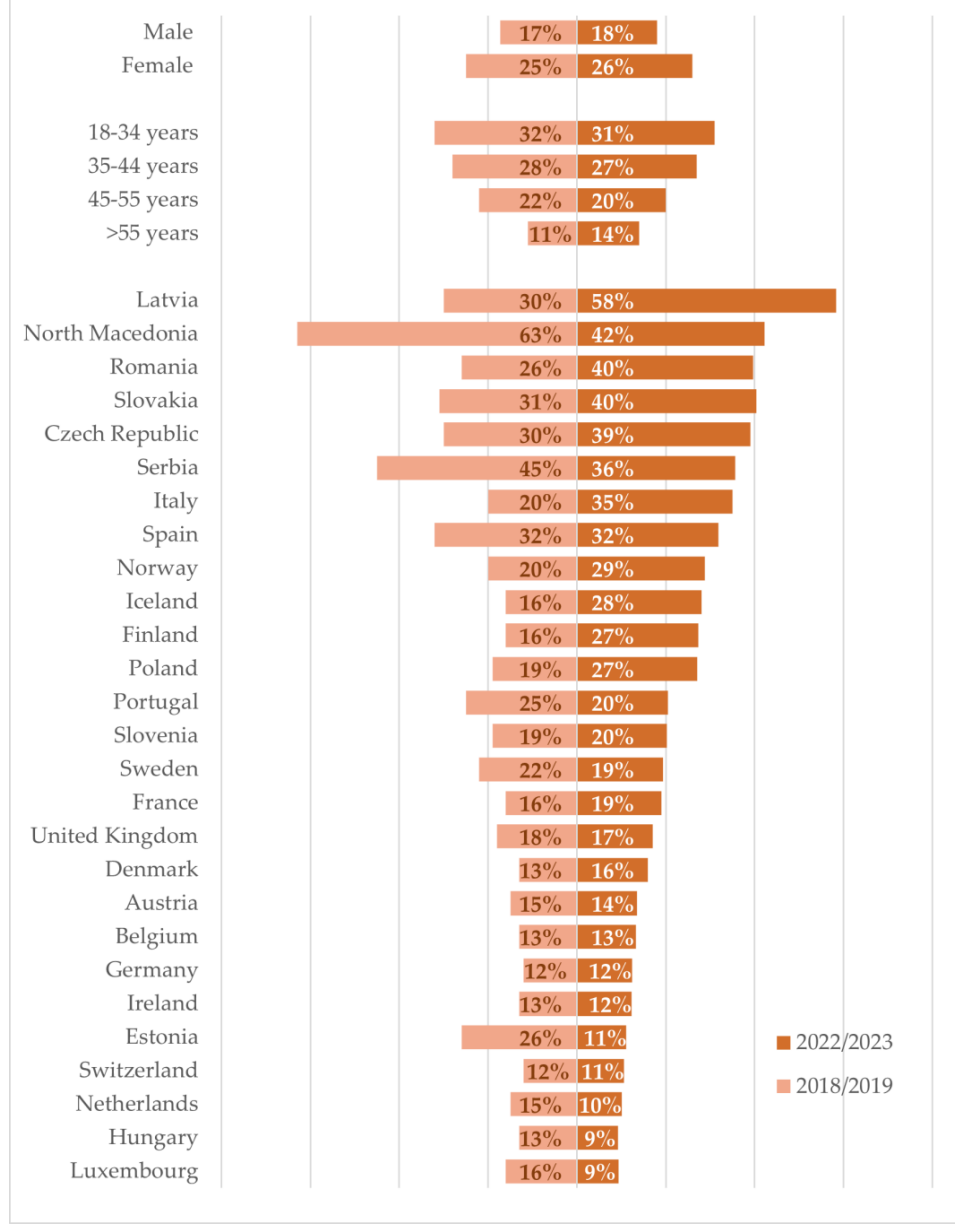
Percentages of veterinarians who took medical leave due to burnout, exhaustion, compassion fatigue, or depression of more than 14 days in the last three years by gender, age, and country.

In total, 22% and 23%, resp., of all veterinarians answering the survey in 2018/2019 and 2022/2023 needed to take more than two weeks off work due to burnout, exhaustion, compassion fatigue, or depression in the last three years. This number varied very much between countries in both surveys, with the lowest numbers reported in Germany and Switzerland (12%, resp.) versus the highest number in North Macedonia (63%) in 2018/2019, and 9% being reported in Luxembourg and Hungary versus Latvia (58%) and North Macedonia (42%) in 2022/2023. It remained consistent over the two surveys that more female veterinarians reported taking medical leave (25% and 26%, resp.) than male veterinarians (17.3% and 18%, resp.), and early-career veterinarians taking more than senior veterinarians (Figure 3). Variations can also be noted within the veterinary profession, with veterinarians working in NGOs (66%) and in food hygiene (31%) reporting the highest proportions in 2028/2019 and in the group ‘telemedicine, consultancy and emergency care’ with 27% in 2022/2023. Consistently, veterinarians from the group ‘education and research’ reported the lowest proportions of medical leave for mental health reasons in both surveys (13% in 2018/2019, 18% in 2022/2023). Veterinarians working in independent practices (23%, *n* = 4743) and corporate practices (24%, *n* = 1385) took equal amounts of medical leave for mental health reasons in 2022/2023, and the frequency of medical leave was lowest in 2022/2023, with 20% in practices with more than 26 veterinarians (full-time equivalents) (data for 2018/2019 not available).

### 3.4. Warwick–Edinburgh Mental Wellbeing Scale

**Figure 4:**
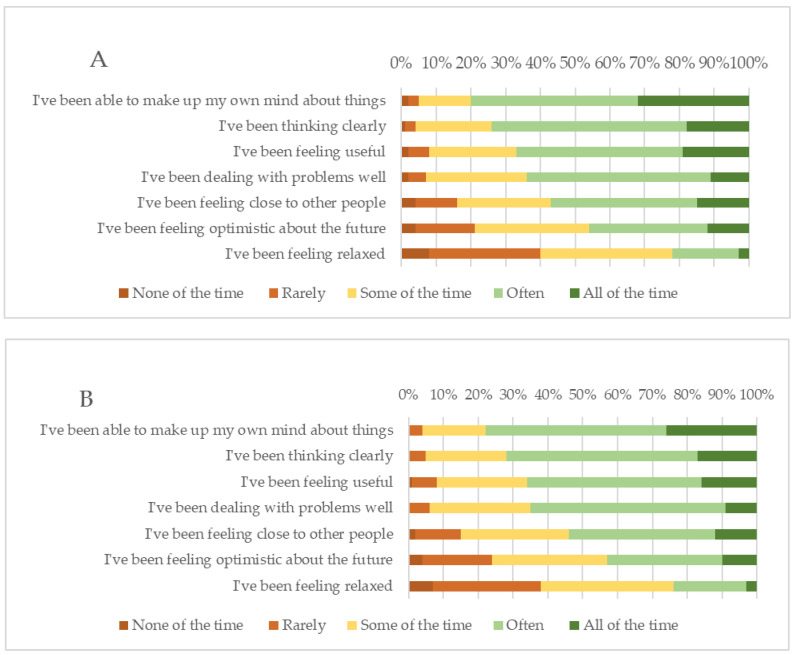
Sum of response frequencies (‘none of the time’, ‘rarely’, ‘some of the time’, ‘often’, ‘all of the time’) (bars) and percentages (*x*-axis) of the seven WEMWBS items in descending order of best scored questions. (Panel **A**): years 2018/2019, (Panel **B**): years 2022/2023.

**Figure 5:**
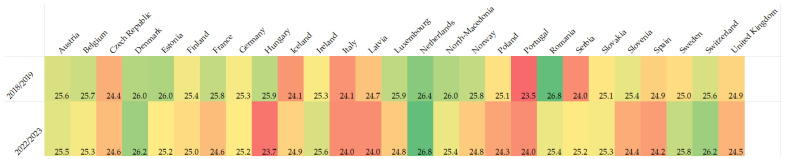
Heatmap of the sum of WEMWB scores per country showing visually the countries with a higher overall WEMWBS score (more positive experiences, thoughts, and feelings) in shades of green and those with lower WEMWB scores in shades of red. (2018/2019: max. 26.80 (green), min. 23.54 (red). 2022/2023: max. 26.80 (green), min. 23.70 (red).

Respondents were asked to fill in the seven-question Warwick–Edinburgh Mental Wellbeing Scale (WEMWBS). WEMWBS asks about experiences, thoughts, and feelings over the past two weeks. Individual items are scored on a five-point Lickert-type scale from 1 (none of the time) to 5 (all of the time), and a total scale score is calculated by summing the seven item scores. These questions asked how often they felt optimistic, relaxed, useful, that they were dealing well with problems, thinking clearly, feeling close to people, and able to make up their mind. The minimum score is 7 and the maximum is 35. After removal of all replies with missing answers, 14, 185 responses (2018/2019) and min. 9704 to max. 9690 responses (2022/2023) were valid. The cumulative score in 2018/2019 was 25, compared to an almost equal cumulative score in 2022/2023 of 24.8. The order of the best-ranked questions remained consistent over the two surveys (Figure 4). In both surveys, wellbeing scores generally increased with age, and females scored lower than males. In 2022/2023, *n* = 6 participants were able to choose ‘other gender’ and scored particularly low, with a sum of 20.8. Hardly any differences were seen between the two surveys for veterinarians working full-time (sum of 25.1 and 24.5, resp.) or veterinarians working part-time (sum of 25.0 and 24.5, resp.). In 2022/2023, a distinction was made between veterinarians working in an independent practice (sum of 24.9, *n* = 4695) and veterinarians working in a corporate practice (sum of 24.3, *n* = 1376), but no significant difference was observed. Consistently over all countries, ‘I’ve been feeling relaxed’ had the highest proportion of ‘none of the time’ and ‘rarely’, with 40% (2018/2019) and 38% (2022/2023) (Figure 5).

## 4. Discussion

Attention to veterinary MWB has increased substantially in the last decade, followed by the development of support programs by many veterinary organizations, companies, and faculties [45]. In our study, we analyzed veterinary MWB based on the results of two representative, consecutive surveys in 2018/2019 and 2022/2023 of the veterinary profession on three indicators: (i) self-reported stress levels, (ii) the need for medical leave due to MWB issues, and (iii) the seven-question Warwick–Edinburgh Mental Wellbeing Scale. Despite multidimensional crises (e.g., the COVID-19 pandemic, inflation, and the war in Ukraine), the overall MWB indicators have remained worryingly high but relatively stable since 2018. While this might sound surprising, other similar studies confirmed that the European average happiness in the COVID-19 years of 2020–2022 was just as high as in the pre-pandemic years of 2017–2019 [46,47]. There was a globe-spanning surge of benevolence in 2020 and especially in 2021, supported by a stronger sense of common purpose, which might have stabilized the perceived situation and been reflected in our results [48]. In both surveys, important differences were spotlighted between countries, with some countries consistently scoring better (e.g., Denmark, The Netherlands) and other countries consistently lower (e.g., Lithuania, Hungary). However, it is acknowledged that the individual MWB of the population in Europe during the pandemic years declined significantly, with higher levels of anxiety and depression and increased demands for mental health services [49]. In addition, various countries and sectors saw a period called the ‘Great Resignation’ during the pandemic years, where many employees who were dissatisfied in their current job position (mostly related to low remuneration, no opportunities for advancement, and feeling disrespected at work) resigned and moved on [50,51,52]. While no precise data are available on the turnover in veterinary workplaces, there are indications that this also happened in the veterinary field, with those who were most unhappy leaving their position within the profession or even leaving the profession completely, and as such, also no longer answering the 2022/2023 survey. Overall, early-career and female veterinarians were identified as being most at risk of an impaired MWB. There are several potential factors to consider that influence these findings. The veterinary profession has evolved, and recent graduates may face different challenges in a changed professional landscape than their more experienced counterparts [53]. Shifts in client expectations, technological advancements, and associated changes in the profession may contribute to additional stress [54]. Recent graduates, regardless of gender, often experience career development pressure to establish themselves in their careers [55]. This pressure can contribute to stress and anxiety, particularly if they perceive that their career progression is slower than expected [56]. Different generations and genders may employ different coping mechanisms. More experienced colleagues may have developed coping strategies over time, while recent graduates may still be learning effective ways to manage stress and challenges [57,58,59]. Societal expectations and gender roles can influence the experiences of female professionals. Women may feel additional pressure to prove themselves in male-dominated fields like veterinary leadership positions, potentially contributing to stress [60]. In addition, gender-based discrimination or bias may affect the experiences of female veterinarians from their undergraduate studies onwards, potentially impacting their mental health [61,62]. Stereotypes and biases can create additional stress and undermine professional confidence [63]. Moreover, young female veterinarians may face challenges in balancing their career and personal life, especially if they are also managing family responsibilities [4,64,65]. This balance can be particularly demanding during early career stages [66]. Furthermore, some countries account for higher proportions of early-career and/or female veterinarians, which might have influenced further the individual country results.

### 4.1. Self-Reported Stress Level

The very nature of veterinary medicine inevitably causes strain due to inherent stressors due to the cumulative responsibility for animal health and welfare, public health, decision making over life and death, economic restrictions on care, and client and societal expectations [67,68,69]. However, avoidable workplace stressors in veterinary medicine, such as an overly high workload, a lack of control over working hours, and high demands, have a serious and direct toll on productivity and the efficiency of work, work quality, and human resources [17]. The culture of veterinary medicine commonly labels veterinarians with adjectives such passionate, hard-working, altruistic, and patient-driven, with expectations for veterinarians to be impervious to stress and fatigue, or beliefs that personal health is subservient to work [70]. These unhealthy norms, blind spots, and tacit assumptions can cause mental and physical stress and unsustainable working conditions [70].

To improve the assessment of stress [71], self-reported stress level ratings on a 4-point Lickert-type scale was applied in 2022/2023, as opposed to a 11-point Lickert-type scale in 2018/2019; however, this impeded direct comparison across the two surveys. Nevertheless, the plotted data do not indicate a drastic change between the two surveys, and compared to other publications, the stress level of veterinarians (6.9 on a 0 (no stress)–10 (highest stress) scale in 2018/2019, and with 57% indicating that they are quite stressed (38%) or very stressed (19%) in 2022/2023) seems consistently higher than in the general population. A global survey from 2020 focusing on veterinarians dealing with companion animals used the same 4-point Lickert-type scale and reported that the least stressed groups are part-time employees, male veterinarians, and professionals with 30+ years of experience. A notable finding regarding the stress levels of European veterinarians showed a sharp increase compared to pre-COVID-19, with an average of 60% of professionals being ‘quite’ or ‘very stressed’, and with the highest stress levels reported from the UK (70% ‘quite’ or ‘very stressed’) and Portugal (87% ‘quite’ or ‘very stressed’) [72]. A study from the American Psychological Association (APA) measured stress levels on a 10-point scale in 2020, with an average of 5.6 based on 2076 adult participants. In the 2022 Gallup report on ‘State of the Global Workplace 2023’, 44% of participants on a global level and 39% on a European level said that they experienced a lot of stress, and full-time employees scored significantly more often at the highest stress level. The Gallup analysis found that work engagement (work involvement, enthusiasm for work) has 3.8 times more influence on employee stress than work location (in the practice, hybrid, or remote work). However, this was for all employees, and no studies are available on whether the same applies for the veterinary profession [73]. Denmark, The Netherlands, and Switzerland remained in the top positions in both surveys, as they do in ours. The same result is shown in other publications, such as the Organization for Economic Co-operation and Development (OECD)’s Better Life Index [74] and the *Journal of Happiness Studies* publication in 2020 on happiness and life satisfaction comparing 21 countries [75,76].

Similarly to our study, the APA data found that different generations experience different levels of stress, with younger adults reporting higher average stress levels (6.1) and stress levels going down with age (ages 56–74; 4.7) [77]. In addition, general mental health was shown to improve with age in the general population too, and was related to improved emotional regulation, communication, and coping skills developing over time [78]. Several other European studies showed identical risk profiles, with stress levels in general being higher in the younger population and females [79,80]. Our results clearly show that early-career and female veterinarians are more likely to have decreased MWB. As identified earlier, female veterinarians have a higher risk of psychological distress and burnout compared to male colleagues, as do early-career veterinarians compared to more senior colleagues [37,38,81,82,83]. Factors that may play a role were identified as a high workload, long working hours, moral distress, ethical dilemmas, difficulty in achieving a good work/life balance, and a lack of support or recognition [35]. In particular, early-career professionals are more likely to face different challenges due to the changed veterinary professional landscape, career development pressure, and still developing coping strategies. Female veterinarians may be more likely to face difficulties due to gender roles and expectations, stereotypes and bias, and managing multiple roles and responsibilities without appropriate support, which can be particularly demanding during early career stages. This can lead to role conflicts, a work–life imbalance, and burnout, which can negatively affect self-esteem, life satisfaction, stress levels, and happiness. It is to be hoped that the next generation of veterinarians will grow up in a society with certain expectations about values, team spirit, a work schedule that also allows for time spent with family and friends, fair remuneration, and possibilities to change to other veterinary work fields or professions [45]. This will demand a culture change within veterinary medicine and veterinary practice, also earlier stated by [15,81], in which a compensation model that incentivizes long hours and promotes heroism is abandoned. This situation is not unique to the veterinary profession, as other healthcare professions face similar challenges. It is therefore essential for all healthcare professions to create supportive and inclusive work environments that are adapted to the needs of the different stages of life and that prioritize the wellbeing of all their employees, regardless of age, gender, or other diversity factors. However, the interpretation of these statistics should also consider survival bias, as burned out professionals are more likely to change careers and be excluded from the sampled population [9].

To address these challenges, many national and regional veterinary organizations worldwide have launched awareness campaigns and begun to implement MWB support programs. Organizations such as Mind Matters International ((MMI), founded by the Royal College of Veterinary Surgeons (RCVS)), Vetlife, and Not One More Vet have been established to raise awareness and improve MWB on an (inter)national level for individuals in need. Despite these programs, it is recognized that not all veterinarians receive the support that they need due to a local lack of availability and stigmatization of MWB issues [12,46].

### 4.2. Medical Leave Due to Reduced Mental Wellbeing

Severe work-related stress is associated with increased risks of long-term medical leave, although affected individuals generally do experience improvements during this time [84,85]. The average number of veterinarians who had taken off two weeks in a row due to burnout, exhaustion, compassion fatigue, or depression in the preceding three years went slightly down from 26% to 23%, although some countries, including Latvia (28%), Italy (15%), and Romania (14%), reported a significant increase. There was a slight decrease in females needing breaks in 2022/2023 (26%) compared with 2018/2019 (25%). According to the OECD, mental illness is one of the main causes of sickness absence and disability in the workforce [86]. However, the exact amount of medical leave taken due to mental illness in Europe is difficult to estimate, as different countries have different definitions, methods, and sources of data collection. According to the latest European Working Conditions Survey (EWCS) in 2020, 9% of people reported having been absent from work for more than 10 days due to health problems in the preceding 12 months. However, EWCS does not distinguish between mental and physical illness [87]. The European Health Interview Survey reported in 2019 that 4.2% of the general population with tertiary education had at least moderate current depressive symptoms [88]. Compared to the general population, German veterinarians were approximately three times more likely to have depression (OR  =  0.349; 95% CI, 0.309 to 0.940) and approximately twice as likely to express current suicidal ideation as the general population sample used (OR  =  0.497; 95% CI, 0.445 to 0.554) [89].

Consistently, early-career and female veterinarians were most in need of a medical mental health break. Large differences can also be observed between countries, ranging from 9% to 58%. Compared to the general EU population, numbers are alarmingly high: 8.7% of women and 5.5% of men aged 15 years and over reported chronic depression in the EU in 2019 [88]. However, these numbers may only be the tip of the iceberg. Moreover, help-seeking behavior may be influenced by stigma, fear of confidentiality issues, and unwanted intervention in healthcare students, practicing veterinarians, and physicians compared to non-healthcare workers, which may prevent appropriate health-related help-seeking behavior [90,91,92]. In our study, we could not access how many had actually sought help, but it is known that veterinarians and other healthcare professionals are systemically disincentivized to acknowledge MWB issues or to seek help due to mental health stigma [93,94] and self-stigma [95]. The difference between veterinarians in need of taking medical leave and the actual rate of taken medical leave remains to be elucidated. For clinical physicians, it was shown that speaking up or seeking help to deal with work-related stress continues to be perceived, especially within the culture of healthcare, as a sign of weakness, despite awareness raising and the known prevalence of burnout [96,97]. In a recent study of veterinarians, the majority (97%) expressed that treatment helped individuals with mental illness lead normal lives, but a much lower proportion (53%) agreed that people were caring and sympathetic toward individuals with mental illness [91]. Negative attitudes toward the presence of social support for mental illness were more likely in veterinarians aged 40–59 years (compared to those aged 20–39 years; 46 vs. 37%, OR = 1.18, *p* < 0.001), in women (compared to men; 75 vs. 64%, *p* < 0.001), by solo practitioners (vs. non-solo; 81 vs. 19%, OR = 1.23, *p* = 0.08), in those with evidence of serious psychological distress (vs. without; OR = 1.55, 22 vs. 9%, *p* < 0.001), and by those reporting mental illness after graduating from veterinary school (vs. reporting no mental illness; 42 vs. 58%, OR = 1.66, *p* < 0.001) [91]. This is also seen in other healthcare professions, where, in a study of 516 university hospital physicians who reported recent suicidal thoughts and/or showed other indicators of psychological ill health, a staggering 78% of these distressed physicians had never sought professional help for depression or burnout [98]. While veterinarians working in research and education reported in our survey the lowest frequencies of medical leave due to mental health issues, a previous study from 2011 of occupational health across various veterinary sectors, those in education and research reported the highest levels of stress, and in another study from 2003, those in research, teaching, industry, and government positions experienced the highest levels of depression [16,25]. Although the situation seems to improve, these numbers are alarming because of the implications for patient care, clinical research, the education of future veterinarians, and for the individual affected veterinarian alike.

### 4.3. The Warwick–Edinburgh Mental Wellbeing Scale

The cumulative WEMBWS score went slightly down from 25 in 2018/2019 to 24.8 in 2022/2023. Compared to a cross-country study in Denmark, the veterinarians in our surveys seem to score similarly compared to the general population, with a Danish general population WEMBWS score in 2016 of 26.4, in Iceland of 25.4 in 2017, and in the UK in 2016 of 22.9 [99]. WEMBWS scoring was undertaken in the UK in 2019, with similar scores (a sum of 47.7/70 for 14-point WEMBWBS), yet there were lower levels of MWB among equine veterinary surgeons and equine veterinary nurses during the COVID-19 pandemic compared to the situation prior to the COVID-19 pandemic [36].

Similarly, respondents in our surveys were consistently positive over both surveys about how often they had been able to make up their own mind and think clearly, but less positive about the future and feeling relaxed [100]. Demographic analysis of the veterinary profession in both surveys showed that early-career professionals and female veterinarians scored lowest. It was shown that a lack of diversity, equity, and inclusiveness in the veterinary profession is frequently reported as a stressor in the UK and USA [101,102,103,104,105]. Timmenga et al. recommended that implementation strategies to increase awareness of MWB and DEI must reach all veterinarians at all levels of their professional careers, e.g., through positive reward programs, campaigns, and webinars, which were universally stated as being very effective in creating awareness and having a large impact [45]. Globally, the number of female veterinarians is increasing and has outpaced the number of male veterinarians in many countries and regions [106,107,108,109,110,111,112,113]. This is also the case in Europe, where the number of female veterinarians is growing rapidly (from 58% in 2018 to 65% in 2023) [2]. Despite this, a gender pay gap is still observed in veterinary practice [2,4], as are client sexism [114], a lack of respect for female practitioners following childbirth and/or taking part-time work [115,116], and gender mismanagement and leadership visibility issues, with females rarely climbing to the upper end of the veterinary hierarchy [109,116,117]. In addition, members of the Lesbian, Gay, Bisexual, Trans, Queer, and Intersex (LGBTQI+) community experience more mental health problems and suicidal ideation in veterinary school and as veterinary professionals [1,118]. A global survey by the International Veterinary Student Association (IVSA) concluded that student discrimination was a serious issue due to their sexual orientation or ethnicity at most veterinary universities [119]. This was underlined by Snyder et al., who were able to show that the veterinary profession in the USA was the least racially diverse (consisting of 93.8% White non-Hispanics), with one of the lowest proportions of people of color compared to other health occupations [120]. While the sample size in our survey of those choosing ‘other gender’ was too small to analyze, the cumulative WEMWBS was strikingly low, and further research is needed to explore the MWB of and specific support for non-cisgender veterinarians. Although DEI support programs were less available, Timmenga et al. recommended that, at the beginning of veterinary training, all students need positive and inclusive role models as well as diverse examples from the veterinary profession [45].

### 4.4. Limitations of the Study

The non-probability snowball sampling of the survey made it difficult to determine the sampling error or generalize inferences about the studied entities based solely on the obtained questionnaire responses. There was a subjective measurement bias in the results, since respondents were asked to rate the stress level on a non-standardized scale and via self-reporting. Medical leave due to burnout, exhaustion, compassion fatigue, or depression may underestimate the true effect of the exposure and the outcome, as other mental health conditions such as anxiety were not adequately reflected, leading to an underreporting of medical leave for mental health conditions. In addition, other factors may be at play than the individual need for medical leave, leading to an inability to actually take the medical leave due to, i.e., stigma or workforce shortages. While the short seven-item WEMWBS is widely used to measure MWB and psychological functioning in individuals, it has limitations in that it is based on self-reporting, there are cultural considerations, and the interpretation is challenging, with no defined threshold for defining good or poor MWB. The accessibility and geographical coverage of the survey were enhanced by providing the questionnaires in different languages. However, even well-translated surveys can be biased by cultural issues. The main considerations were the acceptability of extreme positive or negative opinions of the assessment scales in various cultures, cross-cultural equivalence, and whether respondents could be biased toward answering questions in ways that were socially acceptable.

## 5. Conclusions

Veterinarian MWB is complex and multifaceted, and impaired MWB can affect anyone, regardless of age, gender, or professional background. Our findings showed that despite multidimensional crises, the need for medical leave due to reduced mental health and MWB scores remained stable, with overall stress levels being consistently high. In particular, our results showed that early-career and female professionals are at higher risk, potentially due to the changed veterinary professional landscape, career development pressure, and professionals still developing coping strategies. Moreover, gender roles and expectations, stereotypes, and bias may play a role, as well as the need to manage multiple roles and responsibilities without appropriate support. All these aspects can be particularly demanding during early career stages. However, our results indicate that part-time work seems beneficial for veterinary MWB. Therefore, improving veterinarian wellbeing will be most successful by creating supportive veterinary workplaces that prioritize wellbeing, staff retention, and pay attention to the work/life balance. Despite many psychometric tools reported in the (veterinary) literature to measure MWB, there is a crucial need to define, in the near future, comparable and consistent standards for MWB assessments in healthcare professions.

## Data Availability

The data that support the findings of this study are available on request from the corresponding author, N.D.B. The data are not publicly available due to containing information that could compromise the privacy of survey participants.

## Supplementary Materials

The following supporting information can be downloaded at https://www.mdpi.com/article/10.3390/vetsci11010048/s1: Table S1: STROBE Statement—checklist of items that should be included in reports of cross-sectional studies, adapted from [40]; Table S2: Checklist for reporting results of internet E-surveys (CHERRIES), adapted from [41]; Table S3: Question and answer formats from of the 3rd VetSurvey; Table S4: Analyzed countries, their total respondents for 2018/2019, registered active veterinarians in 2022, participation rate, population, and veterinarians per 1000 population in 2022.
