# Supplementary material for: Veterinarian—Chasing A Dream Job? A Comparative Survey on Wellbeing and Stress Levels among European Veterinarians between 2018 and 2023
Source: Vet Sci. 2024 Jan 22;11(1):48. doi: 10.3390/vetsci11010048 (PMC10820604; doi:10.3390/vetsci11010048)
Supplement: Supplementary file 1 [file vetsci-11-00048-s001.zip › vetsci-2770408-supplementary.pdf]

Supplementary Materials: Veterinarian—Chasing a Dream Job? A Comparative Survey on Wellbeing and Stress Levels among European Veterinarians between 2018 and 2023

Wiebke Jansen, Lizzie Lockett, Tricia Colville, Mette Uldahl and Nancy De Briyne

Table S1: STROBE Statement—checklist of items that should be included in reports of cross-sectional studies, adapted from [40].

	Item No	Recommendation	Page No
Title and Abstract	1	(a) Indicate the study's design with a commonly used term in the title or the abstract	1
		(b) Provide in the abstract an informative and balanced summary of what was done and what was found	1
		Introduction	
Background/ Rationale	2	Explain the scientific background and rationale for the investigation being reported	2
Objectives	3	State specific objectives, including any prespecified hypotheses	2
		**Methods**	
Study design	4	Present key elements of study design early in the paper	2
Setting	5	Describe the setting, locations, and relevant dates, including periods of recruitment, exposure, follow-up, and data collection	3
Participants	6	(a) Give the eligibility criteria, and the sources and methods of selection of participants	3
Variables	7	Clearly define all outcomes, exposures, predictors, potential confounders, and effect modifiers. Give diagnostic criteria, if applicable	N/A
Data Sources/ Measurement	8 *	For each variable of interest, give sources of data and details of methods of assessment (measurement). Describe comparability of assessment methods if there is more than one group	N/A
Bias	9	Describe any efforts to address potential sources of bias	N/A
Study Sze	10	Explain how the study size was arrived at	3
Quantitative Variables	11	Explain how quantitative variables were handled in the analyses. If applicable, describe which groupings were chosen and why	3
		(a) Describe all statistical methods, including those used to control for confounding	3
		(b) Describe any methods used to examine subgroups and interactions	N/A
Statistical Methods	12	(c) Explain how missing data were addressed	N/A
		(d) If applicable, describe analytical methods taking account of sampling strategy	N/A
		(e) Describe any sensitivity analyses	N/A
		**Results**	

		(a) Report numbers of individuals at each stage of study—eg numbers potentially eligible, examined for eligibility, confirmed eligible, included in the study, completing follow-up, and analyzed	3/4
Participants	13 *	(b) Give reasons for non-participation at each stage	N/A
		(c) Consider use of a flow diagram	N/A
Descriptive Data	14 *	(a) Give characteristics of study participants (eg demographic, clinical, social) and information on exposures and potential confounders	Tab. S4
		(b) Indicate number of participants with missing data for each variable of interest	N/A
Outcome Data	15*	Report numbers of outcome events or summary measures	4-8
		(a) Give unadjusted estimates and, if applicable, confounder-adjusted estimates and their precision (eg, 95% confidence interval). Make clear which confounders were adjusted for and why they were included	N/A
Main Results	16	(b) Report category boundaries when continuous variables were categorized	N/A
		(c) If relevant, consider translating estimates of relative risk into absolute risk for a meaningful time period	N/A
Other Analyses	17	Report other analyses done—eg analyses of subgroups and interactions, and sensitivity analyses	N/A
		**Discussion**	
Key Results	18	Summarize key results with reference to study objectives	8
Limitations	19	Discuss limitations of the study, taking into account sources of potential bias or imprecision. Discuss both direction and magnitude of any potential bias	11
Interpretation	20	Give a cautious overall interpretation of results considering objectives, limitations, multiplicity of analyses, results from similar studies, and other relevant evidence	8-10
Generalizability	21	Discuss the generalizability (external validity) of the study results	11
		**Other information**	
Funding	22	Give the source of funding and the role of the funders for the present study and, if applicable, for the original study on which the present article is based	12

* Give information separately for exposed and unexposed groups.

Table S2: Checklist for reporting results of internet E-surveys (CHERRIES), adapted from [41].

Checklist Item	Explanation	Page Number
**Describe Survey Design**	Describe target population, sample frame. Is the sample a convenience sample? (In "open" surveys this is most likely.)	**3**
**IRB Approval**	Mention whether the study has been approved by an IRB.	**12**
**Informed Consent**	Describe the informed consent process. Where were the participants told the length of time of the survey, which data were stored and where and for how long, who the investigator was, and the purpose of the study?	**3, Tab. S3**
**Data Protection**	If any personal information was collected or stored, describe what mechanisms were used to protect unauthorized access.	**N/A**
**Development and Testing**	State how the survey was developed, including whether the usability and technical functionality of the electronic questionnaire had been tested before fielding the questionnaire.	**2**
**Open Survey Versus Closed Survey**	An "open survey" is a survey open for each visitor of a site, while a closed survey is only open to a sample which the investigator knows (password-protected survey).	**2**
**Contact Mode**	Indicate whether or not the initial contact with the potential participants was made on the Internet. (Investigators may also send out questionnaires by mail and allow for Web-based data entry.)	**2**
**Advertising the Survey**	How/where was the survey announced or advertised? Some examples are offline media (newspapers), or online (mailing lists – If yes, which ones?) or banner ads (Where were these banner ads posted and what did they look like?). It is important to know the wording of the announcement as it will heavily influence who chooses to participate. Ideally the survey announcement should be published as an appendix.	**2**
**Web/E-mail**	State the type of e-survey (eg, one posted on a website, or one sent out through e-mail). If it is an e-mail survey, were the responses entered manually into a database, or was there an automatic method for capturing responses?	**2**
**Context**	Describe the Web site (for mailing list/newsgroup) in which the survey was posted. What is the Web site about, who is visiting it, what are visitors normally looking for? Discuss to what degree the content of the Web site could pre-select the sample or influence the results. For example, a survey about vaccination on a anti-immunization Web site will have different results from a Web survey conducted on a government Web site	**2/3**
**Mandatory/Voluntary**	Was it a mandatory survey to be filled in by every visitor who wanted to enter the Web site, or was it a voluntary survey?	**2**
**Incentives**	Were any incentives offered (eg, monetary, prizes, or non-monetary incentives such as an offer to provide the survey results)?	**2**
**Time/Date**	In what timeframe were the data collected?	**2**
**Randomization of Items**	To prevent biases items can be randomized or alternated.	**2**

**or Questionnaires**		
**Adaptive Questioning**	Use adaptive questioning (certain items, or only conditionally displayed based on responses to other items) to reduce number and complexity of the questions.	**3**
**Number of Items**	What was the number of questionnaire items per page? The number of items is an important factor for the completion rate.	**3**
**Number of Screens (Pages)**	Over how many pages was the questionnaire distributed? The number of items is an important factor for the completion rate.	**3**
**Completeness Check**	It is technically possible to do consistency or completeness checks before the questionnaire is submitted. Was this done, and if "yes", how (usually JAVAScript)? An alternative is to check for completeness after the questionnaire has been submitted (and highlight mandatory items). If this has been done, it should be reported. All items should provide a non-response option such as "not applicable" or "rather not say", and selection of one response option should be enforced.	**N/A**
**Review Step**	State whether respondents were able to review and change their answers (eg, through a Back button or a Review step which displays a summary of the responses and asks the respondents if they are correct).	**3**
**Unique Site Visitor**	If you provide view rates or participation rates, you need to define how you determined a unique visitor. There are different techniques available, based on IP addresses or cookies or both.	**IP addresses**
**View Rate (Ratio of Unique Survey Visitors/Unique Site Visitors)**	Requires counting unique visitors to the first page of the survey, divided by the number of unique site visitors (not page views!). It is not unusual to have view rates of less than 0.1 % if the survey is voluntary.	**N/A**
**Participation Rate (Ratio of Unique Visitors who Agreed to Participate/ Unique First Survey Page Visitors)**	Count the unique number of people who filled in the first survey page (or agreed to participate, for example by checking a checkbox), divided by visitors who visit the first page of the survey (or the informed consents page, if present). This can also be called "recruitment" rate.	**N/A**
**Completion Rate (Ratio of Users who Finished the Survey/Users who Agreed to Participate)**	The number of people submitting the last questionnaire page, divided by the number of people who agreed to participate (or submitted the first survey page). This is only relevant if there is a separate "informed consent" page or if the survey goes over several pages. This is a measure for attrition. Note that "completion" can involve leaving questionnaire items blank. This is not a measure for how completely questionnaires were filled in. (If you need a measure for this, use the word "completeness rate".)	**N/A**
**Cookies Used**	Indicate whether cookies were used to assign a unique user identifier to each client computer. If so, mention the page on which the cookie was set and read, and how long the cookie was valid. Were duplicate entries avoided by preventing users access to the survey twice; or were	**N/A**

	duplicate database entries having the same user ID eliminated before analysis? In the latter case, which entries were kept for analysis (eg, the first entry or the most recent)?	
**IP Check**	Indicate whether the IP address of the client computer was used to identify potential duplicate entries from the same user. If so, mention the period of time for which no two entries from the same IP address were allowed (eg, 24 hours). Were duplicate entries avoided by preventing users with the same IP address access to the survey twice; or were duplicate database entries having the same IP address within a given period of time eliminated before analysis? If the latter, which entries were kept for analysis (eg, the first entry or the most recent)?	**N/A**
**Log File Analysis**	Indicate whether other techniques to analyze the log file for identification of multiple entries were used. If so, please describe.	**3**
**Registration**	In "closed" (non-open) surveys, users need to login first and it is easier to prevent duplicate entries from the same user. Describe how this was done. For example, was the survey never displayed a second time once the user had filled it in, or was the username stored together with the survey results and later eliminated? If the latter, which entries were kept for analysis (eg, the first entry or the most recent)?	**2**
**Handling of Incomplete Questionnaires**	Were only completed questionnaires analyzed? Were questionnaires which terminated early (where, for example, users did not go through all questionnaire pages) also analyzed?	**3**
**Questionnaires Submitted with an Atypical Timestamp**	Some investigators may measure the time people needed to fill in a questionnaire and exclude questionnaires that were submitted too soon. Specify the timeframe that was used as a cut-off point, and describe how this point was determined.	**No**
**Statistical Correction**	Indicate whether any methods such as weighting of items or propensity scores have been used to adjust for the non-representative sample; if so, please describe the methods.	**No**

Table S3. Question and answer formats from of the 3rd VetSurvey.

**Q1. Do you consent to take part in this survey?**				
Yes, I consent and am happy to take part in this survey				
No, I do not consent and do not wish to take part in this survey				
**Q2. Please describe yourself. Are you …**				
Male	Female	Other gender category	Prefer not to say	
**Q3. And how old are you?**	years	Do not know	N/A	Prefer not to say
**Q4. In what country do you currently practice veterinary medicine?**				
Albania				
Austria				
Belgium				
Bosnia and Herzegovina				
Bulgaria				
Croatia				
Cyprus				
Czech Republic				
Denmark				
Estonia				
Finland				
France				
Germany				
Greece				
Hungary				
Iceland				
Ireland				
Italy				
Latvia				
Lithuania				
Luxembourg				
Malta				
Montenegro				
Netherlands				
North Macedonia				
Norway				
Poland				
Portugal				
Romania				
Russian Federation				
Serbia				
Slovakia				
Slovenia				
Spain				
Sweden				
Switzerland				
Turkey				
Ukraine				

United Kingdom
Other country (please specify)

Q5. How many years have you worked as a veterinarian? Note: Working as veterinarians means all areas of work in which you use your veterinary qualification. So, it includes clinical practice, but also working in government, in academia, in (food, animal health) industry, veterinary consultancy, corporate offices and many other sectors.

years	N/A	Refusal	Do not know

Q6. Which of the following best describes your current employment?

Private veterinary practice	Independent veterinary practice
Corporate veterinary practice/part of a chain of practices	Telemedicine
Consultancy only	Emergency care only
Public service/government	Education-research
Industry (pharmaceutical, food, agricultural etc)	Food production (meat, dairy, fish)
Feed production for animals	Pharma human
Pharma vet	Medical devices
Quality assurance	Other services to the veterinary profession
NGO/Charity	Other - as a veterinarian
Outside the veterinary profession	

Q7. How stressed are you?

Very stressed	Quite stressed	A little bit stressed	Not at all stressed

Q8. In the last three years, did you need to take a break for more than two weeks due to depression, burn-out, exhaustion or compassion fatigue (Holidays excluded)?

Yes	No

Q9. Below are some statements about feelings and thoughts. Please select the option that best describes your experience of each over the last 2 weeks.

I've been feeling optimistic about the future	Never	Rarely	Some of the time	Often	All of the time
I've been feeling useful	Never	Rarely	Some of the time	Often	All of the time
I've been feeling relaxed	Never	Rarely	Some of the time	Often	All of the time
I've been dealing with problems well	Never	Rarely	Some of the time	Often	All of the time
I've been thinking clearly	Never	Rarely	Some of the time	Often	All of the time
I've been feeling close to other people	Never	Rarely	Some of the time	Often	All of the time
I've been able to make up my own mind about things	Never	Rarely	Some of the time	Often	All of the time

Table S4. Analyzed countries, their total respondents for 2018/2019 and 2022/2023, registered active veterinarians in 2022, participation rate, population and veterinarians per 1000 population in 2022.

**Country**	**Total Respondents**		**Registered active veterinarians in 2022** (ex. retired, unemployed and not working)	**Partici-pation rate**	**Population (Wordometer_UN data on 1 August 2023)**	**Veterinarians per 1000 population**
	2018/ 2019	2022/ 2023				
**Austria**	212	278	3,373	8%	8,958,960	0.38
**Belgium**	211	411	6,179	7%	11,686,140	0.53
**Czech Republic**	393	308	4,476	7%	10,495,295	0.43
**Denmark**	385	266	2,650	10%	5,910,913	0.45
**Estonia**	81	56	854	7%	1,322,765	0.65
**Finland**	266	233	2,000	12%	5,545,475	0.36
**France**	1,323	1231	21,000	6%	64,756,584	0.32
**Germany**	1,618	1310	32,930	4%	83,294,633	0.40
**Hungary**	139	116	3,142	4%	10,156,239	0.31
**Iceland**	31	30	182	16%	375,318	0.48
**Ireland**	368	253	2,900	9%	5,056,935	0.57
**Italy**	1,182	767	29,424	3%	58,870,762	0.50
**Latvia**	64	49	751	7%	1,830,211	0.41
**Luxembourg**	82	58	295	20%	654,768	0.45
**Netherlands**	259	170	6,000	3%	17,618,299	0.34
**North Macedonia**	75	79	428	18%	2,085,679	0.21
**Norway**	425	246	2,815	9%	5,474,360	0.51
**Poland**	464	317	14,005	2%	41,026,067	0.34
**Portugal**	1,267	1493	6,829	22%	10,247,605	0.67
**Romania**	848	580	6,402	9%	19,892,812	0.32
**Serbia**	144	122	2,541	5%	7,149,077	0.36
**Slovakia**	294	384	2,317	17%	5,795,199	0.40
**Slovenia**	155	183	1,035	18%	2,119,675	0.49
**Spain**	2,641	1322	26,549	5%	47,519,628	0.56
**Sweden**	406	540	3,643	15%	10,612,086	0.34
**Switzerland**	104	246	2,751	9%	8,796,669	0.31
**United Kingdom**	453	625	27,448	2%	67,736,802	0.41
**EUROPE (27)**	13,890	11,673	212,919	5%	514,988,956	0.41
